# Implementation of Ebola Case-Finding Using a Village Chieftaincy Taskforce in a Remote Outbreak — Liberia, 2014

**Published:** 2015-02-27

**Authors:** José E. Hagan, Wilmot Smith, Satish K. Pillai, Kristin Yeoman, Sundeep Gupta, John Neatherlin, Laurence Slutsker, Kim A. Lindblade, Kevin M. DeCock, Francis Kateh, Tolbert Nyenswah

**Affiliations:** 1 Epidemic Intelligence Service, CDC; 2 Center for Global Health, CDC; 3 Rivercess County Health Team, Liberia; 4 National Center for Emerging and Zoonotic Infectious Diseases, CDC; 5 National Institute for Occupational Safety and Health, CDC; 6 Ministry of Health and Social Welfare, Liberia.

On October 16, 2014, a woman aged 48 years traveled from Monrovia, Liberia, to the Kayah region of Rivercess County, a remote, resource-poor, and sparsely populated region of Liberia, and died on October 21 with symptoms compatible with Ebola virus disease (Ebola). She was buried in accordance with local tradition, which included grooming, touching, and kissing the body by family and other community members while it was being prepared for burial. During October 24–November 12, eight persons with probable and 13 with confirmed Ebola epidemiologically linked to the deceased woman had onset of symptoms. Nineteen of the 21 persons lived in five nearby villages in Kayah region; two, both with probable cases, lived in neighboring Grand Bassa County (Figure). Four of the confirmed cases in Kayah were linked by time and location, although the source case could not be determined because the patients had more than one exposure.

On November 9, the Rivercess County Health Team requested assistance from CDC, Médecins Sans Frontières, the World Health Organization, and other partners to assess area needs and guide response efforts. Initial public health actions from November 11 to November 17 included health promotion messaging, rapid construction and staffing of a temporary isolation and treatment facility, and an investigation that included case finding, area mapping, and interviews with village leaders and community representatives. Village leaders reported that some known contacts had fled to the surrounding forest, raising concerns that Ebola might have been transmitted to surrounding villages. Many of these villages lack cellular phone connectivity and are only reachable by footpaths through dense forest. In addition, movement between neighboring village communities is common and is unrestricted by administrative boundaries, which facilitates the possibility of wide dispersion of contacts who might not all be included in contact listing. For these reasons, traditional contact tracing (1) was determined to be inadequate to stop transmission. This report describes a novel system to supplement contact tracing by quickly identifying potential cases among villages in a remote area with limited infrastructure.

An active surveillance network was created by working with community leadership to establish village-to-village communication relays that could, with minimal additional investment of resources or training, rapidly overcome the obstacle to outbreak control that was posed by the lack of means of communication and transportation in the remote area. The Kayah region is a chiefdom containing 37 villages with approximately 5,000 persons, led by a council of village chiefs. The CDC and county health team requested a meeting with the council of chiefs, as well as representatives of men, women, youths, and elders of the community, to hear their concerns, identify needs, and to engage them as partners in social mobilization to sensitize the community to the urgency of outbreak control, and to overcome resistance to Ebola messaging and measures to prevent transmission. Following this meeting, the chiefs and community members expressed a high level of enthusiasm for participating in the Ebola response in a concrete and visible way as members of a Chieftancy Task Force, and contributed to the design of the surveillance system.

Capitalizing on existing political and public health structures, the county health team and CDC created a process for active case-finding at the village level and reporting of cases and deaths to the district health team. The Kayah chiefdom includes a single health clinic, and is organized into four community health clusters of five to 14 neighboring villages per cluster. Each cluster is represented by a community health committee in the principal (largest and most accessible) village of the cluster. Within each cluster a maximum of 2 hours of walking was required to reach the nearest village or cellphone-accessible area. All cases in Rivercess County occurred in the Gozohn community health cluster. In each of the 11 villages in this cluster, Task Force members identified a person (when available, this was a general community health volunteer, a village member trained in general community health issues) to perform a daily survey of all village households to identify persons who were feeling unwell for any reason and any deaths from any cause. Special attention was paid to subjective fever, severe body pain or weakness, or gastrointestinal symptoms, the most common early signs and symptoms of Ebola (2). A report of any of these findings was transmitted daily to a community health committee representative, either by telephone or in person.

Reports from the villages were expected at least every 2 days, even if zero cases or deaths were detected. A simple log of reports was kept by the community health committee representative and the district health team to identify villages with missed reports and record basic details of positive reports. The district health team followed up each alert with case investigation or triage, as appropriate. Missed reports were followed up by reestablishing contact with the village by telephone or in person, as needed.

Surveillance was implemented on November 17 in Gozohn village. It was expanded to the rest of the Gozohn community health cluster on November 20, and continued until 21 days following the last possible exposure to a person with confirmed or suspected Ebola in the community, a total of 16 days. There was a high level of acceptance by village surveillance volunteers and community members. Reports were filed from all 11 villages every 2 days; one suspected case and one death were detected, both of which were determined not to be Ebola by laboratory test or verbal autopsy, respectively. Following cessation of daily reporting, village and community health cluster representatives expressed interest in scaling down the system to continue passive reporting of suspected Ebola cases. Widespread prominent display of Ebola health promotion posters on houses and in common areas suggested that active participation by village leadership and community representatives was helping to raise awareness and acceptance of Ebola prevention messaging.

This system could be easily used or adapted for use in other remote areas where there is concern about active Ebola transmission and unidentified contacts, and where local geography and lack of communication and transportation infrastructure impede case-finding and contact tracing from a more central administrative level (e.g., district or county level). Adequate performance of the system can be measured using indicators such as the number of missed reports and the number of cases that are only detected from other sources (e.g., patients admitted to an Ebola treatment unit or cases reported directly to the district or county surveillance teams). In addition, if implemented on a scale that includes multiple community health clusters and larger population sizes, a well-performing system should detect all-cause mortality in the surveillance catchment area of at least the baseline crude death rate for Liberia, up to one per 10,000 population per day, the expected crude mortality rate in complex humanitarian emergency settings (3).

## Figures and Tables

**FIGURE:**
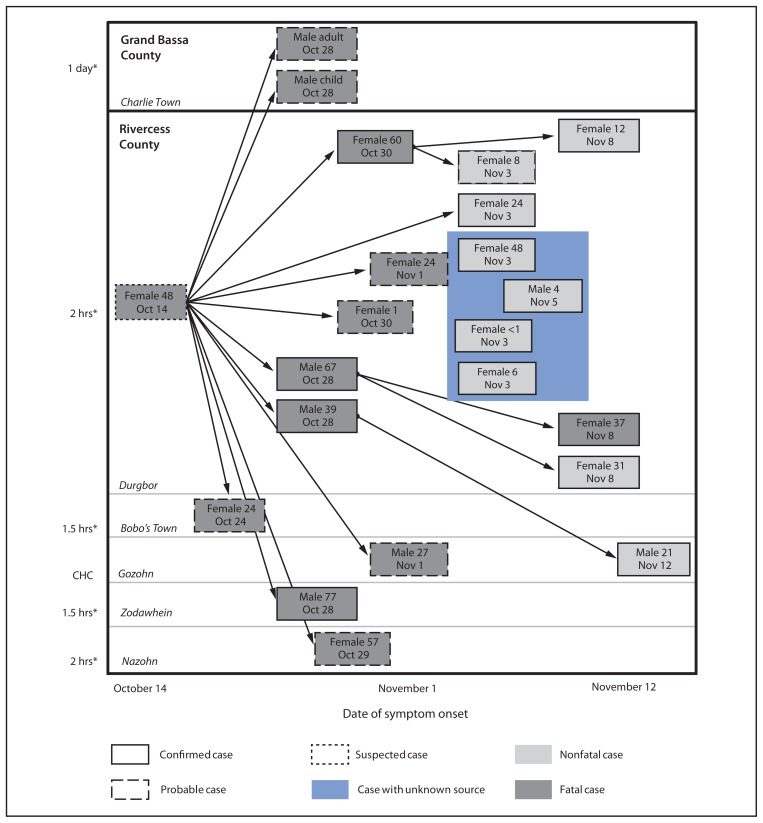
Ebola cases epidemiologically linked to the death of a woman aged 48 years, by patient’s sex, age in years, and date of symptom onset — Rivercess and Grand Bassa counties, Liberia, October 14–November 12, 2014 * Approximate walking distance from Gozohn, the location of the community health committee (CHC) that serves the affected villages in Rivercess County.
